# A rapid screening tool for psychological distress in children 3–6years old: results of a validation study

**DOI:** 10.1186/1471-244X-12-170

**Published:** 2012-10-16

**Authors:** Caroline Marquer, Caroline Barry, Yoram Mouchenik, Sarah Hustache, Douma M Djibo, Mahamane L Manzo, Bruno Falissard, Anne Révah-Lévy, Rebecca F Grais, Marie-Rose Moro

**Affiliations:** 1 Epicentre, 8 rue St Sabin, Paris, F-75011, France; 2 Inserm U669, Université Paris-Sud et Université Paris Descartes, Paris, France; 3 Université Toulouse le Mirail, Toulouse, France; 4 Hospital Sainte-Anne, Paris, France; 5 National program of mental health, Ministry of Health, Niamey, Niger; 6 Regional Department of Public Health, Maradi, Niger; 7 Médecins Sans Frontières, Paris, France; 8 Université Paris Descartes, Sorbonne Paris Cité, Hôpital Cochin, Assistance Publique Hôpitaux de Paris, Paris, France

## Abstract

**Background:**

The mental health needs of young children in humanitarian contexts often remain unaddressed. The lack of a validated, rapid and simple tool for screening combined with few mental health professionals able to accurately diagnose and provide appropriate care mean that young children remain without care. Here, we present the results of the principle cross-cultural validation of the “Psychological Screening for Young Children aged 3 to 6” (PSYCAa3-6). The PSYCa 3–6 is a simple scale for children 3 to 6 years old administered by non-specialists, to screen young children in crises and thereby refer them to care if needed.

**Methods:**

This study was conducted in Maradi, Niger. The scale was translated into Hausa, using corroboration of independent translations. A cross-cultural validation was implemented using quantitative and qualitative methods. A random sample of 580 mothers or caregivers of children 3 to 6 years old were included. The tool was psychometrically examined and diagnostic properties were assessed comparing the PSYCa 3–6 against a clinical interview as the gold standard.

**Results:**

The PSYCa 3–6 Hausa version demonstrated good concurrent validity, as scores correlated with the gold standard and the Clinical Global Impression Severity Scale (CGI-S) [rho = 0.41, p-value = 0.00]. A reduction procedure was used to reduce the scale from 40 to 22 items. The test-retest reliability of the PSYCa 3–6 was found to be high (ICC 0.81, CI95% [0.68; 0.89]). In our sample, although not the purpose of this study, approximately 54 of 580 children required subsequent follow-up with a psychologist.

**Conclusions:**

To our knowledge, this is the first validation of a screening scale for children 3 to 6 years old with a cross-cultural validation component, for use in humanitarian contexts. The Hausa version of the PSYCa 3–6 is a reliable and a valuable screening tool for psychological distress. Further studies to replicate our findings and additional validations of the PSYCa 3–6 in other populations may help improve the delivery of mental health care to children.

## Background

The mental health needs of young children in humanitarian contexts often remain unaddressed [1-4]. During the acute phase of a humanitarian emergency, and in humanitarian contexts in general, psychological care of children may come far down on the list of priorities. The limited number of both local and international medical professionals combined with the relative lack of mental health professionals in these settings also hinders the implementation of mental health activities [5]. Further, even when mental health professionals are present, they are rarely specialists in young children. Psychological distress in young children is particularly difficult to evaluate by non-specialists, requiring knowledge of normal child development, as many behaviors are normal at certain ages but not at others. Young children, between 3 and 6 years old, are in a vulnerable psychological period, which can have consequences on the quality of their emotional, cognitive, and physical capacities [6,7]. Although the psychological response of children depends on their individual, family, and social environments among many other factors, recognizing the diversity of potential psychological responses is essential to provide appropriate interventions [8-10]. The training and infrastructure needs in children’s mental health remain the ultimate goal, but in their absence, tools that help identify children who require further assessment would help to rationalize scare resources and orient children to care in humanitarian contexts.

The lack of cross-culturally valid instruments, and data about child psychological difficulties, is a public health concern in humanitarian contexts [2,11-14]. Although scales exist for general psychological difficulties, none of them concern children aged 3 to 6 years in humanitarian contexts [15-17]. Further, before use, screening tools should be cross-culturally validated for specific contexts [11,18-20]. Difficulties with mental health assessment include lack of consistent assessment tools for measuring psychological distress, lack of cross-cultural validation research and variation in methods for validity testing and differences in methods of translation [21]. Typically, examining mental health cross-culturally involves simply transposing Western assessment tools with no examination of their validity [22]. As result, children remain unscreened or evaluated using a scale not designed for either the specificities of childhood psychological distress or the context. The lack of a validated, rapid and simple tool for screening, combined with few mental health professionals able to accurately diagnose and provide appropriate treatment, mean that young children may remain without appropriate care.

In addition, interest in humanitarian settings has focused primarily on trauma rather than other disorders or psychological difficulties [1,12,23-25], adding also that most of such studies were implemented in conflict affected settings. This focus is based on the assumption that exposure to violence frequently entails post-traumatic symptoms [12,26]. This premise has been criticized recently, as well as the use of only a post-traumatic scale for screening [11,27]. Research addressing the cross-cultural validity of Western diagnostic classification of psychological difficulties in such contexts remains essential to ensure appropriate care is provided [11,28-31]. Recent research has shown the importance of tools able to detect and orient children in need, but has focused on children older than 6 years with an emphasis on post-traumatic stress disorder [22,32-35]. In addition to PTSD, recent studies have also shown the importance of addressing depression and anxiety disorders [35].

Our aim was to begin to respond to one of the gaps in addressing the mental health needs of young children in humanitarian contexts. Although there are many valid models of screening, evaluation and care, the need for a simple, rapid screening scale administered by non-specialists would fill one of the many gaps in responding to the mental health needs of young children in humanitarian contexts. We report the results of a study to cross-culturally adapt and assess the reliability, validity and psychometric properties of the Psychological Screening for Young Children aged 3 to 6 (PSYCa 3–6) following cross-cultural validation. Although the PSYCa 3–6 had been validated in several populations [36,37], it had never undergone but had never undergone a rigorous cross-cultural validation process.

The entire validation process for the PSYCa 3-6-22 includes three steps, one called principal, including a large sample, and two called secondary, implemented to strengthen the results. The principle validation will be presented here. The overall process including the three steps will be the purpose of another publication. Selection of study sites were based on the political context and the population of children expected to be exposed to conflict to facilitate the evaluation of the post-traumatic component of the screening scale.

## Methods

### Setting

The principal validation took place in the rural region of Maradi, located in the south of Niger along the Nigerian border. This population is mainly Hausa speakers living in villages interspersed with Fulani and Touareg camps.

### Study population

The rationale for the selection of this population included the language itself as well as the prevalence of psychological distress. First, Hausa is one of the principle languages of Sub-Saharan Africa, spoken by an estimated 30 to 50 million people. Although common language does not imply common culture, by selecting a commonly spoken language, the possibility of the use of the scale improved. Second, we expected a lower prevalence of post-trauma in this Hausa population than in other in post-conflict contexts. At the time of the study, the political environment was stable we could consider that young children had not been exposed to countrywide conflict. This was an important factor as the tool should be useful not only in situations of conflict or post-conflict. Niger faces recurrent food insecurity and malnutrition is chronic [38,39]. Validation of a tool in Hausa, covering multiple registers, could be useful in this context. Finally, existing partnerships with the Ministry of Health of Niger provided the possibility of wide-scale use of the tool within the health system after validation. This was an important consideration providing the possibility of improving the mental health care of children in Niger.

#### Tool

The “Psychological Screening for Young Children aged 3 to 6” (PSYCa 3–6) is an indicative tool, not designed for diagnosis, but rather to determine a general level of psychological distress in children 3 to 6 years old. It is a hetero-questionnaire completed by the parent or caregiver through an interviewer. Unlike existing generalist tools, the PSYCa 3–6 includes a psycho-traumatic component. Data collected through the PSYCa 3–6 allows for the inferences of psychological difficulties and their main register of expression: depression, phobia, anxiety, regression, psychosomatic complaints, and post-traumatic disorder. The PSYCa 3–6, in its initial version, included 40 items concerning child behavior in the form of easy questions caregivers answer by never/not at all, or sometimes/a few times or often/frequently/always. For example, “Does your child have bad dreams or nightmares often?” Each interviewer, which may be non-specialists, read the questions and score depending to the response. At the end of administration, responses are summed to compute a score ranging from 0 to 80 with higher scores indicating greater distress. Developed in French in 1999 for Albanian and Kosovar child refugees in Macedonia, the tool has been subsequently refined and improved [37,40]. The results of a preliminary validation in France on 52 children, showed promising results but no formal validation of the scale has been conducted [36] [Table 1.

**Table 1:** Reduced version of PSYCa 3–6 – 22

**The modality of response was 0, 1 or 2 (never, sometimes, often) for each item**			
0	1	2	1. The child stutters
0	1	2	2. The child refuses to eat repeatedly
0	1	2	3. The child wakes up frequently, insomnia
0	1	2	4. The child is absent, seems somewhere else or in “his world”, has difficulties to interact with you
0	1	2	5. The child had a bad dream or a nightmare that comes often
0	1	2	6. The child is frightened, worried, anxious
0	1	2	7. The child has difficulty to be clean (pee, poop)
0	1	2	8. The child refuses to separate with one of his parents, siblings etc.
0	1	2	9. The child eats too much
0	1	2	10. The child does not speak or very little, his language is very different from children of his age
0	1	2	11. The child refuses to eat certain foods and chooses what to eat at every meal
0	1	2	12. The child has difficulty falling asleep
0	1	2	13. The child has outbursts, have uncontrolled movements for no apparent reason
0	1	2	14. The child complains of pain or complains about his body without obvious medical reason
0	1	2	15. The child is unable to sit still, he moves constantly
0	1	2	16. The child refuses to leave the household
0	1	2	17. The child is tired, discouraged
0	1	2	18. The child's behavior is really too aggressive, he is violent (at home and / or outside)
0	1	2	19. The child isolates himself or often moves away from others
0	1	2	20. The child is easily overwhelmed by his emotions anger, sadness fraternal jealousy etc.
0	1	2	21. The child plays repetitive games or activities
0	1	2	22. The child runs away or avoids sounds, images or specifics situations

### Adaptation and translation

The adaptability of the tool was discussed with experienced mental health professionals and anthropologists originating from Niger or who had done research in the country. A linguistic and cultural translation of the tool was performed from French to Hausa. Ideally, several bilingual persons (same fluency in the two languages and experience with mental health instruments) are used to obtain translations and blind back translation [41,42]. Here, two professional translators (Hausa/French) translated the tool independently. The two resulting versions were compared and the few differences were resolved. The translated tool was then administered to a small group of caregivers in a pilot phase for acceptance, adequacy and applicability [43]. A list containing misunderstood items developed and an alternate proposed translation was made. The final version was fixed by the two translator’s consensus taking into account differences between spoken and written Hausa.

Focus group discussions with community key informants, individual interviews and expert meetings were conducted during adaptation and translation phase. Focus groups helped to elucidate understanding of the beliefs and perceptions about child development and expectations of the outcome of treatment [44,45]. They were also conducted to ensure understanding of each of the scale’s items and enhance the quality of the clinicians’ diagnosis during the validation phase (see methods) [46] and ensure that misclassification was minimized [47-49]. Variations in clinical presentation of psychological distress may lead to misidentification. Cultural variations, social interactions and specific contextual factors vary cross-culturally, and might influence the experience, representation, expression of psychological difficulties [50]. Consultations with psychiatrists and anthropologists and the health care staff of the hospitals in Niamey (the country’s capital) and Maradi to define developmental milestones and child psychopathology were also organized.

### Training

All interviewers were selected by meeting the main criteria, experience in questionnaire administration, fluent in Hausa and French and without a background in mental health. They were trained over a three-day period on how to administer the questionnaire. After a general presentation of the tool, items were presented and discussed one by one. Role-plays were used to simulate interviews and training provided on the information and informed consent procedure. Interviewers were also trained on study exclusion criteria. The exclusion criteria for interviewers were based on visible, recognizable difficulties and interviewers were trained on the basics of mental retardation and the severe classifications of the ICD-10. A one-day pilot phase followed the theoretical training to assure standardization of administration and reinforce the theoretical training. The translator participated in the interviewer’s training and also performed an additional day of training on the translation process itself [51]. Interviewers were supervised during the duration of data collection to respond to any difficulties or questions.

### Sampling

The study population included a random sample of 580 caregivers recruited between November 2009 and July 2010. A comprehensive information and awareness campaign was organized before study implementation to inform the population about the aims and objectives of the study. Caregivers were selected using cluster-based sampling, with population proportional to size weighting, a standard methodology in populations where population data is either limited or inaccurate [52]. Within the region, 68 villages or city districts were randomly chosen (22 districts in Maradi city, and 23 villages each in the districts of Madarounfa and Guidan Roumdji) [53]. The random sample in the villages followed the traditional method by spinning a pen, as random starting direction from a central location in the cluster. Households lying on this transect from the center to the border of the cluster were counted and then one of them was chosen at random. Proximity selection was then used to select subsequent households as the "next nearest" until the desired sample size was reached [52,54].

In each household, caregivers needed to have at least one child aged 3 to 6 years old, be Hausa speakers, and resident in the study area (Maradi city, Madarounfa, Guidan Roumdji). As mothers were the most knowledgeable of the daily activities and behaviors of children, they were asked to respond to the questionnaire. Children identified by the mother/caregivers as presenting a mental disorder, mental retardation, development disorder, and/or psychosis were excluded. Interviewers also excluded children who met visible, recognizable criteria of mental retardation or grave development disorders. These children were referred immediately for free care. All mothers and caregivers were read an information letter describing the study and asked for written informed consent before enrollment.

### Study procedures

The first phase of the study consisted of administration of the tool to 325 mothers and caregivers. The aim of this phase was to collect data to examine the psychometric properties of the tool, reliability and test-retest reliability before proceeding further with the validation (see data analysis). A total of 201 children completed the PSYCa 3–6 once to evaluate internal consistency, 51 completed the PSYCa 3–6 twice for temporal stability, and 51 completed the PSYCa 3–6 twice with two different interviewers for interrater reliability. During the morning, each interviewer included 7 to 8 children meeting the inclusion criteria. At midday, the interviewers met and exchanged children. Each interviewer had to provide all needed information for his colleague to find the children selected. Interrater reliability was measured by re-administering PSYCa3-6 independently on the same day at different times by two interviewers. The timeframe of re-administration was the same. The day started including 7 to 8 children, after administration, caregivers were given an appointment for the same day in the afternoon with the same interviewer.

**Figure 1:**
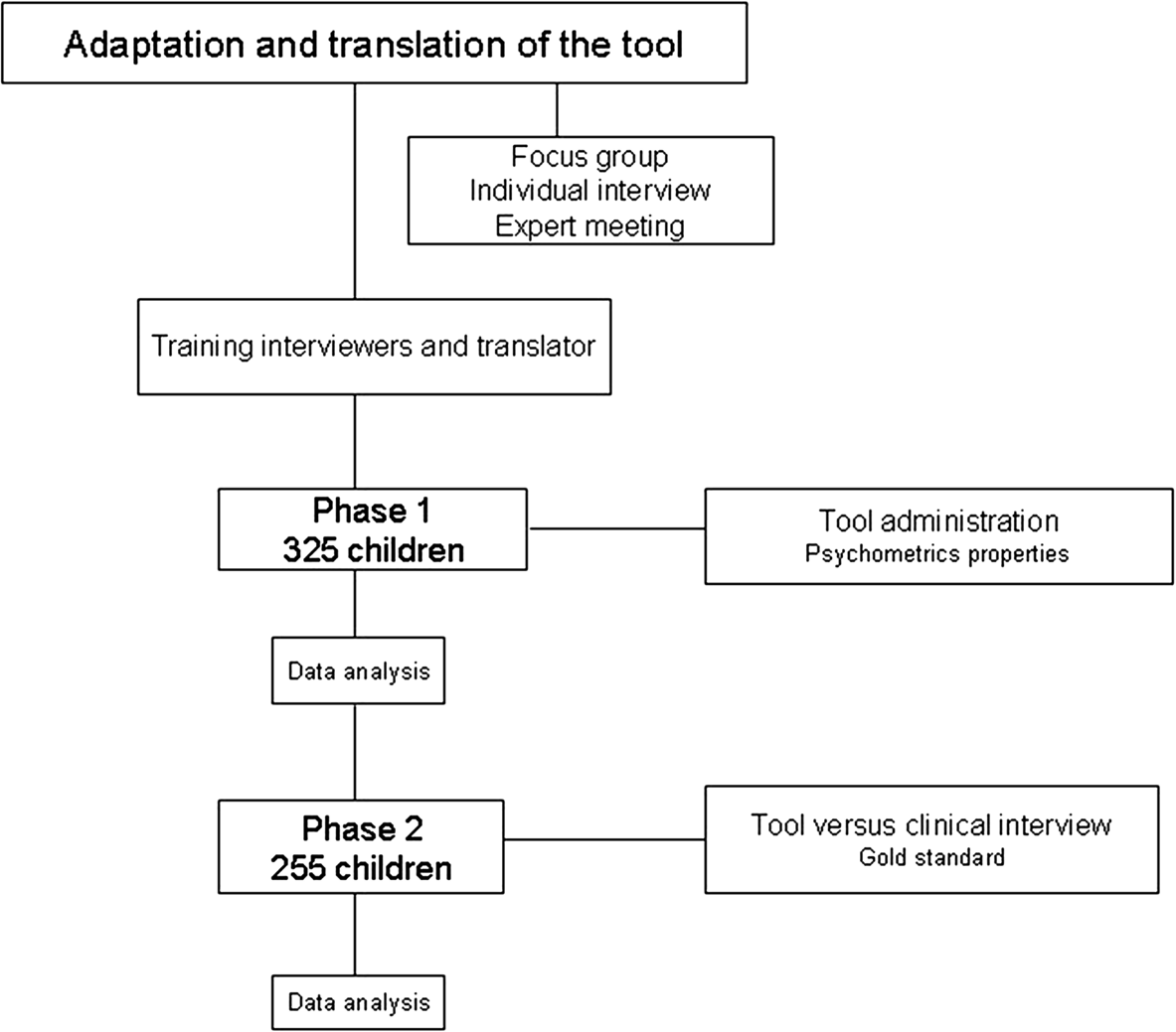
Study flow-chart.

In the second phase of the study, the interviewer administered the tool and this was followed by a clinical evaluation as the gold standard for 255 children. The psychologist completed the Clinical Global Impression-Severity Scale (CGI-S), a seven-point severity scale, assessing a patient’s current symptom severity, and answered the question “does the child need psychological/psychiatric care?”. The CGI-S is used widely in medical care and clinical research because of its face validity and practicability with the same wording irrespective of the pathology [55,56]. The clinician also completed a semi-structured clinical interview, and performed a diagnosis based on ICD-10 classification [33,57].The psychologist was under the supervision of a senior clinical psychologist. In case of psychopathological disorders, specific psychological care consisting of individual based care were provided at home. Mental health care providers in the area were informed of the study and were aware of any referrals for additional care [Figure 1.

Two clinical psychologists carried out the individual interviews (CM, YM), trained in child development and cross-cultural psychology. Each individual interview was a direct and confidential. For each interview, the translator was present. Clinical psychologists were blinded to the score of the tool (administered immediately prior).

### Data analysis

First, using data collected in phase I, psychometric properties were analyzed. We performed a descriptive analysis (missing data, scatter-plot of responses, floor and ceiling effects) and redundancy (estimation of Pearson’s correlation coefficient between items two by two).

Internal consistency of the PSYCAa3-6 was evaluated by Cronbach’s alpha for the entire scale. Test-retest and interrater reliability were estimated using intra-class correlation coefficients (ICC). Kappa statistics were used to calculate the degree of interrater agreement for each question.

Second, in phase II, external validity was tested on 255 children by comparing the reported PSYCa 3–6 scores against the clinical psychologists’ evaluation, answering to the question “Does the child need psychological/psychiatric care?”.To strengthen the results, PSYCa 3–6 total scores were correlated with CGI-S scores. Receiver Operating Characteristic (ROC) curve analyses were performed to evaluate the PSYCa 3–6 screening properties and determine an optimal cut-off. Kruskal-Wallis tests were used to assess the association between PSYCa 3–6 scores and each child's socio-demographic characteristics. The cut off for of 17 was based on prior use of the scale prior to its validation.

Third, an expert panel consisting of a child psychiatrist, research psychologists and a statistician reviewed the results and discussed item reduction; which items should remain, be further modified, and those to be removed (item reduction and clarification). Criteria for removal was based on the clinical importance of the item in this population and in others, the number of items covering the same clinical domain, readability, relevance, redundancy, and on psychometric criteria (floor effect, inter-item correlation, reliability and concurrent validity). We used R software (version 2.10.0) for all analyses. Item reduction and the factor analyses will be presented in a forthcoming manuscript.

### Ethical considerations

The protocol was submitted for approval to the National Consultative Ethics Committee of Niger, Ministry of Public Health of Niger and the Committee for the Protection of Persons (CPP) Ile de France XI. After informed consent was obtained, caregivers were asked to answer the questionnaire. The consent process included two documents, an information sheet and consent form, both translated in Hausa. Patient data were confidential unless it was deemed necessary to protect the health of the patient. The electronic database contained no names or addresses of patients to ensure their anonymity. Children requiring psychological care received appropriate and free care by a clinical psychologist. The PSYCa 3–6, once validated, will be made available to all health actors who wish in Niger.

## Results

In total, 580 children participated in the study. From December 2009 to February 2010, 325 children (160 males (49.2%) and 165 females (50.8%), mean age 52 months) were included. A total of 201 children completed the PSYCa 3–6 once to evaluate internal consistency, 51 completed the PSYCa 3–6 twice for temporal stability, and 51 completed the PSYCa 3–6 twice with two different interviewers for interrater reliability. From May 2010 to July 2010, 255 children were included for the evaluation of diagnostic properties (123 males (48.2%) and 132 girls (51.8%), mean age 52 months) [Table 2]. The item reduction procedure yielded a 22-item instrument (scores 0–44) and results are presented as follows.

**Table 2:** Characteristics of children included in the study, Maradi, Niger, 2010

**Socio-demographics (n=580)**		
**Gender**		
Girls	297	(51.8%)
Boys	283	(48.8%)
**Age (months)**		
36-47	214	(36.9%)
48-59	194	(33.4%)
60-72	172	(29.7%)
**Live most of the time with**		
Both parents	491	(85.2%)
One parent	42	(7.3%)
Someone else	43	(7.5%)
Go to school*	358	(62.0%)
**Number of siblings** ‡	4	(3-6)
**Number of deceased siblings** ‡	1	(0–2)

Data are median (IQR) or numbers (%).

* school, nursery school or day nursery.

‡ same father and same mother.

In the population of 580 children, the proportion of missing data or “I don’t know” response per item was low (average 1.6%, <5% for all but 4 items) suggesting good acceptability and that it was easy to complete. With the exception of item 21 (“moves constantly”), the distribution of answers was left-skewed with a higher floor rather than ceiling effect reflecting the low traumatic exposition of this sample. The mean PSYCa 3–6 scores were 7.13 (SD = 3.95, interquartile range 4–9, min 0 max 27). There was no significant difference between the PSYCa 3–6 scores of boys and girls (p = .16), or between children 36, 48, or 60 months of age (p = 0.5) but scores on the PSYCa 3–6 were significantly higher for children who were detected by their own family as having difficulties such as fears, outbursts, sleep disorders, recurrent nightmares problem and were oriented to a traditional practitioner (p = 0.0002).

The test-retest reliability of the PSYCa 3–6 was found to be high (ICC 0.81, CI9% [0.68; 0.89]). Interrater reliability was satisfactory (ICC of 0.69, CI 95% [0.45; 0.80]). The tool demonstrated acceptable internal consistency (Cronbach’s alpha 0.61, CI 95% [0.54; 0.65] for a screening tool validation including several dimension (depression, anxiety, post traumatic component) [20,58].

**Figure 2:**
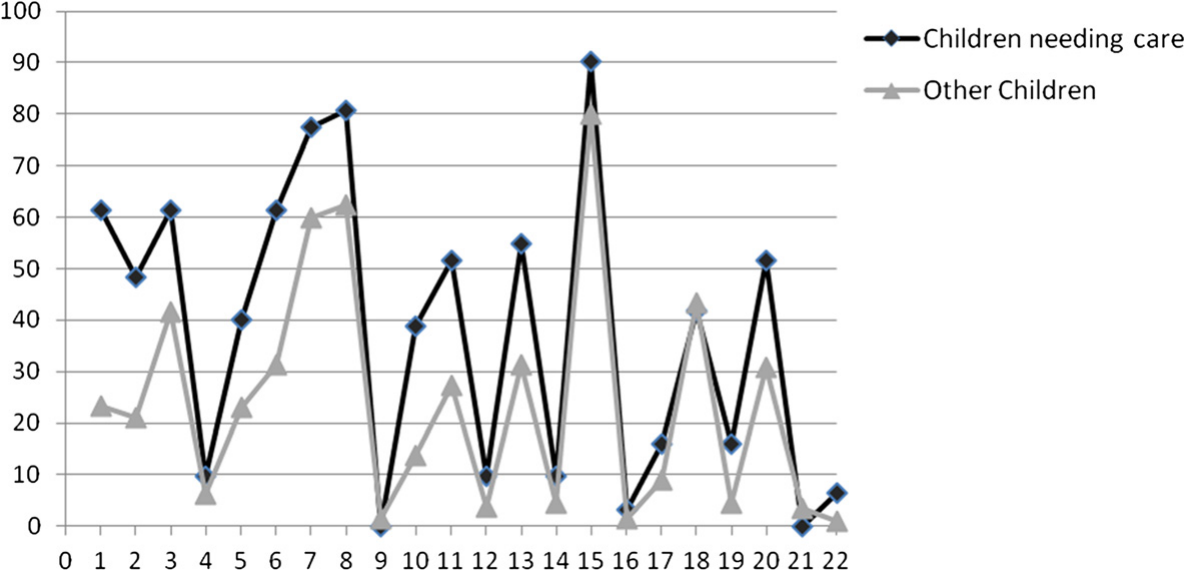
Frequency of positive responses (sometimes, often) between children needing care and others.

**Figure 3:**
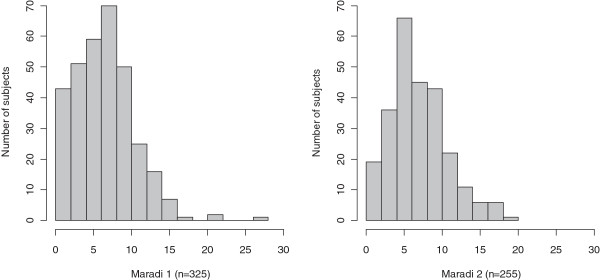
Histograms for PSYCa 3–6 score.

**Figure 4:**
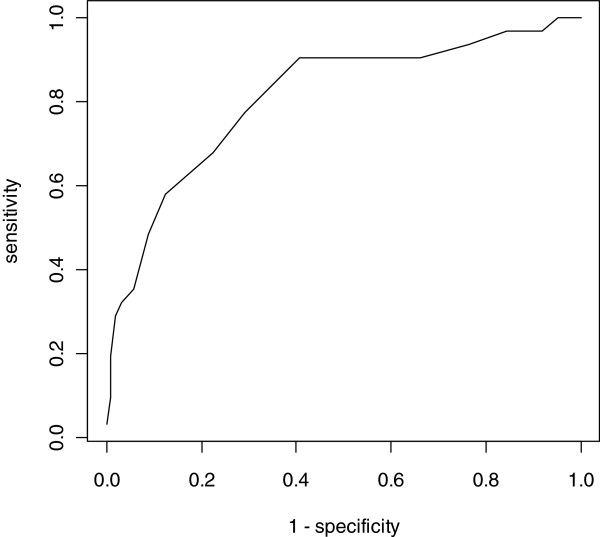
Receiver operating characteristics (ROC) curve of the PSYCa 3–6.

During the second phase, psychologists identified 31 children in need of psychological orientation and 224 children not requiring care. Concerning the CGI-S scale among these 255 children, 187 (73.3%) were rated as 1 “normal, not at all ill”, 29 (11.4%) with borderline CGI, 23 (9%) mildly ill, 12 (4.7%) moderately ill, 4 (1.6%) markedly ill. None were rated as severely or extremely ill. Overall, the 255 children evaluated by psychologist had a mean CGI-S of 1 [1; 3] for the children without orientation and 4 [2; 5] for children oriented towards additional assessment (p < 0.001). The PSYCa 3–6 exhibited significant positive correlations with CGI severity scale (rho = 0.41, p-value = 0.0001). Mean PSYCa 3–6 scores were significantly higher among the 31 children requiring care than those that did not (p-value = 0.0001 [Table 3). The frequency of positive responses to twenty-two items in children requiring care and in other children is shown Figure 2. The ROC analysis resulted in an area under the curve of 0.81 (CI 95% [0.73, 0.89]) [Figures 2, 3 and 4. When the cut-off value of PSYCa 3–6 was nine according to the point closest to the upper left-hand corner of the graph, the sensitivity and specificity were 0.77 and 0.71, respectively [59].

**Table 3:** PSYCa 3–6 scores comparisons between groups

**Clinical interview (n=255)**	PSYCa 3–6 score*	**p-value‡**
**CGI**		<0.0001
normal	6.53 (3.28)	
borderline	8.28 (3.17)	
mildly ill	10.17 (3.85)	
moderately ill or more	12.56 (4.34)	
**Does the child need psychological/psychiatric care?**		<0.0001
No	6.86 (3.35)	
Yes	11.58 (4.27)	

* mean (SD).

‡ Wilcoxon rank sum test.

## Discussion

To our knowledge, this is the first validation of a screening scale for children aged 3 to 6 years old, with a cross-cultural validation component, for use in humanitarian contexts. The PSYCa 3–6 enables the rapid screening of children and can be administered by non-specialists. The results of this study suggest that the Hausa version of the PSYCa 3–6 is a reliable and a valuable tool for screening psychological distress in children 3 to 6 years old. The tool was acceptable to caregivers and interviewers (after training and piloting). The sample of patients for this validation was large compared to other validation studies further strengthening our results [13,37,42,60].

Screening tools provide an important means to facilitate addressing the mental health needs of children in humanitarian emergencies and contexts. Difficulties of young children may remain unnoticed or undetected by both local and international health actors [61] and may have short and/or long term developmental consequences [8,45,62,63]. Screening children provides a means to identify those in need of additional evaluation and eventual care as well as recognizing the limited resources available in these contexts. The reduction from a 40 to 22-item scale also provides additional encouragement that the scale could be administered to an often large number of children in a relatively short period of time. The simple administration of the screening scale also provides additional awareness and understanding of the overall status of the population for health care workers addressing the situations. In humanitarian contexts, a part from the acute phase of an emergency, the tool could also be used to identify difficult to reach children (due to either distance or isolation for example) and refer only those in need of additional evaluation to health structures. However, it is important to highlight that this tool allows for the identification of children requiring further evaluation, but the lack of mental health professionals remains. As with all public health interventions, identification of children in need does not unfortunately always follow with their receipt of appropriate care. Greater investments are needed to ensure that children mental health needs are addressed, and certainly that they are only screened if appropriate care is available [64,65].

We would like to highlight two key points for discussion, especially relevant for future validation studies. First, the translation process was long and involved a linguistic specialist, anthropologist, psychiatrist and psychologist. We first attempted a classical translation/back translation procedure [13,42,66-70], but due to significantly differences concerning written and spoken Hausa, we used independent translations resolved by discussion and pilot testing.

Second, due to the lack of previously cross-cultural validated scales in Hausa, we chose to use a classic individual interview by a psychologist as our gold standard [47,69,70]. Two major types of assessment of mental disorders are used in epidemiological studies: semi structured clinical interviews and lay-administered structure diagnostic questionnaires [71]. To date, there has not been agreement the most appropriate validation method for global mental health research with children [22,42,72,73]. Although use of a clinical interview appeared here the most rigorous choice in this context as opposed to using another tool. The psychologist, trained in cross-cultural psychology and mental health care in children based their diagnosis on ICD-10 classification. To strengthen the clinical interviews, qualitative research concerning child development, child rearing, psychological difficulties in Hausa culture was conducted [35]. Concordance between the clinical interview and the PSYCa 3–6 suggest that use of the ICD-10 did not influence the results presented here.

An apparent limitation is that we do not present a traditional psychometric validation. The traditional validation process (item analysis, factor analysis, etc.) of a psychometric tool was developed in the absence of a gold standard [74]. The objective of the PSYCa 3–6 is to screen subjects who need further evaluation for psychiatric/psychological care. In an ideal situation, the clinician decides, after an interview, if such a care is required. Our gold standard was the clinician’s answer to the question: “does the child need psychological/psychiatric care?”. For this reason, we validated the PSYCa 3–6 as compared with the above question and classical statistics. Most other screening tools in medicine are based on the same methodology; this is the case for example for scores of gravity in intensive care units such as the APACHE score [75], validated against mortality rather than with psychometric tools. After secondary evaluations have been completed, the factor structure of the PSYCa 3–6, corresponding to different clinical conditions should be investigated. The internal consistency may be viewed as a limitation from a psychometric perspective. However, as the PSYCa 3–6 is a screening tool for psychological difficulties, the scale is not one-dimensional to ensure the detection of psychological difficulties in several area of psychopathology. As the PSYCa 3–6 includes several domains, this is not unexpected.

An additional limitation concerns the test-retest and interrater reliability. Both were estimated from interviews performed on the same day. Time between the different interviews is problematic since short interval are prone to recall bias, while long intervals risk being associated with the clinical evolution of the subject evaluated.

Finally, we used initially a cut-off of 17, based on the results of previous use of the scale [36,40]. This number was calculated on 40 items, the initial version, scoring up to 80 (2 points per item). As previously documented, cut-off scores established with Western child populations are not necessarily comparable in others settings [27,43]. After reduction of the scale and analysis, we refined the cut-off to 8/9. The cut-off requires further analysis in subsequent validations to assure stability in other cross-cultural contexts.

## Conclusions

The results of this first validation show promising results, suggesting that the PSYCa 3–6 should be validated in other contexts, to test the post-traumatic component of the tool and to simplify further the instrument. From a public health standpoint, the ability to identify potential psychological difficulties in young children represents a significant advancement for the possibility of addressing children’s’ mental health needs in difficult contexts [76]. Additional efforts to adapt and validate simple screening scales for use in humanitarian contexts should be encouraged.

## Competing interests

The authors declare that they have no competing interests.

## Author contributions

CM coordinated the implementation of the study, participated in the interpretation of the results and drafted the manuscript. CB performed the statistical analysis and. YM participated in the design of the study and in revising the manuscript. SH participated in the design of the study and revised the final manuscript. MDD and MML participated in the design of the study and revising the manuscript. BF participated in the design of the study, statistical analysis, interpretation of the results and revising the manuscript. ARL provided interpretation of the results and revised the manuscript. RFG and MRM conceived the study and the protocol, participated in its design and implementation and revised the manuscript. All authors read and approved the final manuscript.

## Funding

This study was funded by Medecins sans Frontieres.

## Pre-publication history

The pre-publication history for this paper can be accessed here:

http://www.biomedcentral.com/1471-244X/12/170/prepub
